# Arrhythmogenic mitral valve prolapse—a systematic review of ventricular arrhythmia and sudden cardiac death outcomes before and after mitral valve surgery

**DOI:** 10.1002/joa3.70108

**Published:** 2025-07-15

**Authors:** James N. Cameron, Nigel Sutherland, Chee Loong Chow, Hui‐Chen Han, Matias Yudi, Rajiv Mahajan, Anand Ganesan, Avi Sabbag, Kristina H. Haugaa, Jai Raman, Prashanthan Sanders, Omar Farouque, Han S. Lim

**Affiliations:** ^1^ Department of Cardiology Austin Health Melbourne Victoria Australia; ^2^ Faculty of Medicine, Dentistry and Health Sciences University of Melbourne Melbourne Victoria Australia; ^3^ Department of Cardiology Northern Health Melbourne Victoria Australia; ^4^ Victorian Heart Institute Monash University Clayton Victoria Australia; ^5^ Adelaide Medical School University of Adelaide Adelaide South Australia Australia; ^6^ Lyell McEwin Hospital Northern Adelaide Health Local Health Network Elizabeth Vale South Australia Australia; ^7^ Department of Cardiovascular Medicine Flinders Medical Centre Adelaide South Australia Australia; ^8^ The Davidai Center for Rhythm Disturbances and Pacing, Chaim Sheba Medical Center Tel Hashomer Israel; ^9^ ProCardio Center for Cardiological Innovation, Department of Cardiology Oslo University Hospital, Rikshospitalet Oslo Norway; ^10^ University of Oslo Oslo Norway; ^11^ Department of Cardiac Surgery Austin Health Melbourne Victoria Australia; ^12^ Centre for Heart Rhythm Disorders, South Australian Health and Medical Research Institute University of Adelaide and Royal Adelaide Hospital Adelaide South Australia Australia

**Keywords:** mitral valve prolapse, mitral valve surgery, percutaneous mitral valve repair, sudden cardiac death, ventricular arrhythmia

## Abstract

**Background:**

Several autopsy and observational studies have investigated the link between mitral valve prolapse (MVP) and sudden cardiac death (SCD) given the well accepted yet rare occurrence of ventricular arrhythmias (VA). Whether surgical intervention for arrhythmogenic MVP (aMVP) reduces VA and SCD risk remains unknown.

**Methods:**

A systematic literature review was conducted using the PubMed database in December 2024. Studies documented in English were included if patients had undergone mitral valve (MV) surgery (MVS; repair or replacement) for MVP with documented rates of VA or SCD pre‐ and postintervention.

**Results:**

Sixteen identified studies (8 cohort and 8 case studies) comprised 1233 patients (receiving medical or surgical treatment) with a pooled mean age of 61.5 years and 41.9% being female. A total of 657 MVP patients underwent MVS. Seven cohort studies reported rates of VA pre‐ and postintervention, with six of these and all case studies reporting a significant reduction. The remaining cohort study reported a reduction in SCD.

**Conclusions:**

This systematic review indicates a reduction in VA following current guideline‐directed MVS for MVP. However, a residual risk of VA and SCD may remain postintervention.

## Graphical abstract

This systematic review aims to determine whether MVS reduces the incidence of VA and SCD in patients with MVP. Available studies show a likely reduction when surgery is performed in line with current guideline‐directed indications; however, a residual risk of VA and SCD remains.

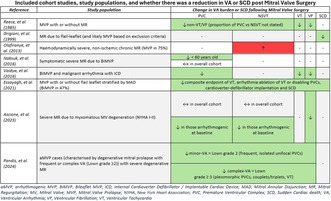

## 1 INTRODUCTION

Several autopsy and observational studies have investigated the link between mitral valve prolapse (MVP) and sudden cardiac death (SCD) due to the well accepted yet rare occurrence of complex ventricular arrhythmias (VA) in this cohort.^1^, ^2^, ^3^, ^4^, ^5^, ^6^, ^7^ Few studies, however, have considered whether arrhythmia burden and, more importantly, SCD are reduced following surgical correction of arrhythmogenic (malignant) MVP (aMVP). Such insights might provide stimulus to consider whether earlier mitral valve (MV) surgery may be warranted outside of currently indicated surgical guidelines.

Although there are numerous independent predictors of long‐term survival after MV surgery (MVS) (replacement or repair), including preoperative left ventricular (LV) and right ventricular ejection fractions (RVEF),^8^, ^9^, ^10^ there is to date limited data regarding the prognostic implications of pre‐ and/or postoperative VA, with SCD continuing to influence long‐term survival post‐MVS.^8^, ^11^, ^12^

MVP is best diagnosed using echocardiography in the parasternal (or apical) long‐axis window, defined as a >2 mm systolic displacement of either mitral leaflet into the left atrium (LA) relative to the mitral annular plane.^13^ In addition to complex VA and SCD, complications of MVP include mitral regurgitation (MR), congestive heart failure (HF), infective endocarditis, and cerebral embolic events. The prevalence of MVP in the general population is approximately 2%–3%,^14^, ^15^ occurring most commonly due to fibromyxomatous changes in one or both leaflets.

A practical difficulty in the management of aMVP is its incidence remaining uncertain. Nishimura in 1985 highlighted the need to stratify patients with MVP for the risk of SCD, reporting a SCD incidence of 16–40 per 10,000 (0.16%–0.4%) per year in 237 minimally symptomatic or asymptomatic patients with MVP.^16^ Basso et al.'s (2001) review of major causes of SCD in patients younger than 40 years (6 studies, 1960–1999) reported MVP in 12% (the third most common finding) of autopsy diagnosed cases.^1^ More recently, we performed a systematic review and meta‐analysis of cases and studies reporting SCD events in patients with MVP, which estimated 217 SCD events per 100,000 person‐years and a yearly SCD incidence of 0.14%, with MVP in 11.7% of SCD cases where the cause remained undetermined.^4^, ^7^

Although MVP's SCD event rate might be less than that in other nonischemic arrhythmogenic syndromes such as hypertrophic cardiomyopathy (HCM), its relative contribution toward SCD in the general adult population is the same, if not greater, due to a significantly higher prevalence.^17^ Using these aforementioned event rates, assuming a 2023 global population of 8.1 billion, 421,848 of the 194.4 million people estimated to have MVP would be at risk of SCD per year.

We present here the first systematic review to our knowledge which investigates whether the incidence of VA and SCD in patients with MVP is reduced following MVS. We investigate and discuss (1) whether there is a reduction in VA associated with MVP after valve surgery, (2) predictors of VA and SCD following intervention, and (3) whether there is scope for current guideline‐directed MVS indications to be extended such that they consider arrhythmic burden in those with aMVP.

## 2 METHOD

### 2.1 Data sources and search strategy

**FIGURE 1:**
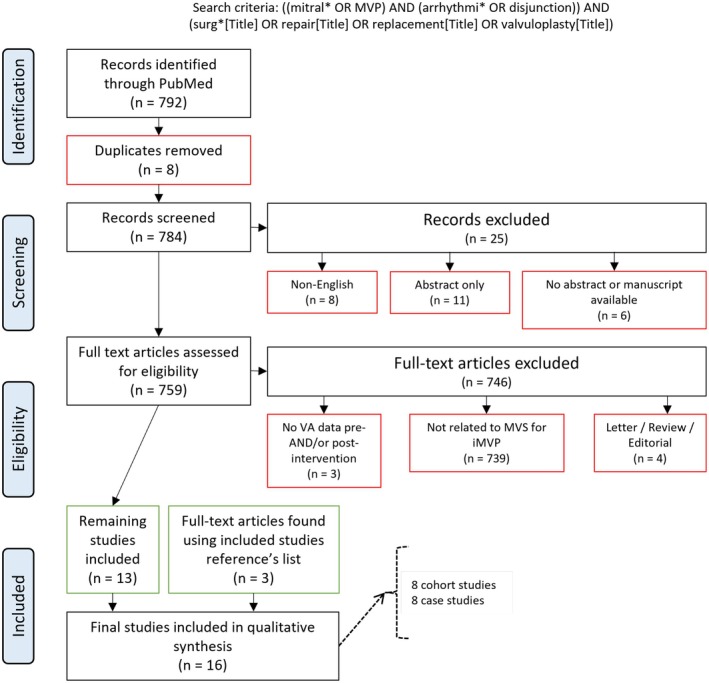
PRISMA search algorithm.

An electronic search of the PubMed database was performed in December 2024 to identify cases of MVP that had undergone surgical management with documented rates of arrhythmias/SCD pre‐ and postintervention. PubMed search terms were ((mitral* OR MVP) AND (arrhythmi* OR disjunction)) AND (surg*[Title] OR repair[Title] OR replacement[Title] OR valvuloplasty[Title]). The full PRISMA study flowchart for our search strategy is shown in Figure 1.

Titles and abstracts were screened for relevance by two reviewers (J.C. and N.S.) who independently conducted the literature search and performed data extraction. Listed references were reviewed to identify additional eligible studies. Data items to be collected were prespecified before conducting the literature search. Identified studies and extracted data were reviewed and verified by a third investigator.

### 2.2 Study selection and data analysis

Included studies met the following criteria: (1) patients had undergone either MVS (MV repair or replacement) for MVP, with (2) documented rates of VA or SCD pre‐ and postintervention, and (3) were reported in English. Reports from case series <5 patients were included for discussion (and included only in Supplemental Results Tables) if individual patient age and sex could be determined, with reports published only in abstract/letter forms excluded. Heterogeneous endpoints precluded formal meta‐analysis.

## 3 RESULTS

### 3.1 Studies

A total of 792 studies were screened with 759 initially deemed eligible for inclusion and full‐text evaluation (Figure 1). Three of these studies were excluded due to lack of VA data pre‐and/or postintervention, 739 did not relate to MVS for MVP, and four were in the form of letters, reviews, or editorials. Three studies were included using references from articles obtained using this search strategy.

Sixteen studies (eight cohort^2^, ^18^, ^19^, ^20^, ^21^, ^22^, ^23^, ^24^ and eight case studies^25^, ^26^, ^27^, ^28^, ^29^, ^30^, ^31^, ^32^) involved patients with MVP undergoing MVS, with one^19^ relating to MR secondary to flail‐leaflet (exclusion criteria being papillary muscle rupture, previous valve surgery, moderate/severe MS or AV disease, congenital heart disease) and therefore likely MVP.

These 16 studies comprised 1233 patients (receiving medical or surgical treatment) with a pooled mean age of 61.5 years and 41.9% being female. A total of 657 MVP patients underwent MVS (surgical method was unspecified in 204 cases). In the included cohort studies, 371 patients underwent MV repair (including two percutaneous repairs with MitraClip), 54 underwent MV replacement, 188 underwent MV surgery where the specific method (repair or replacement) was not specified, and 37 patients were included where procedural details were variably reported (33 annuloplasty ± unspecified repair, 4 commissural plication), highlighting the inconsistencies in the reporting of source data.

Included cohort studies, descriptions of each study population, length of follow‐up, trial design, and whether there was a reduction in VA or SCD post MVS are summarized in Tables 1 and 2. All other comprehensive details of the included studies are presented in Tables S1–S6.

**TABLE 1:** Included cohort studies with descriptions of each study population, trial design, diagnostic criteria, and methodology.

References	Study design	Study population	Total number (% female)	Mean age (years)	Number undergoing MVS (repair/replacement/unspecified)	Diagnostic criteria and methodology
Reece, et al. (1985)^23^	Prospective cohort study analysis	MVP with or without MR	37 (70%)	46.7	37 (0/0/37)	Prior documented MVP (and confirmed on preoperative cardiac catheterisation) Normal coronary arteries, no other cardiac pathological condition except that related to the MV Postoperatively, contacted by letter/telephone to complete questionnaire regarding residual or new symptoms and status
Grigioni, et al. (1999)^19^	Prospective cohort study analysis	MR due to flail‐leaflet (and likely MVP based on exclusion criteria)	348 (26%)	67 ± 12	186 (0/0/186)	Echocardiogram Clinical records, telephone calls, postal surveys, examination of death certificates and autopsy records Sudden death defined if it occurred within 1 h of being previously medically stable
Olafiranye, et al. (2013)^21^	Prospective cohort study analysis	Hemodynamically severe, nonischemic chronic MR (MVP in 75%)	57 (53%)	58 ± 12	57 (22/35/0)	Verified by catheterisation Annual 24 h ambulatory ECGs, 2‐D and Doppler echocardiography Rest and exercise radionuclide cineangiography 1‐year post‐MVS
Naksuk, et al. (2016)^20^	Retrospective cohort study analysis and an individual case study	Symptomatic severe MR due to BiMVP	32 (53.1%)	63.5 ± 12.8	32 (30/0/2)	Serial ECGs, pre‐ and postoperative Holter monitoring Those with ICDs were identified and reports interrogated
Vaidya, et al. (2016)^24^	Retrospective series of cases	BiMVP and malignant arrhythmia	5 (60%)	51 M, 51F, 57F, 55F, 58 M	5 (4/1/0)	Echocardiography or description by the surgeon at the time of surgery ICD interrogation
Essayagh, et al. (2021)^2^	Prospective cohort study analysis	iMVP with or without flail leaflet stratified by MAD (BiMVP in 47%)	595 (47%)	61 ± 16	183 (170/13/0)	Clinical and doppler echocardiographic evaluation at diagnosis, including symptoms, clinical history, and comorbidities Holter recordings reviewed by an electrophysiologist blinded to clinical, echocardiographic, and outcome data
Ascione, et al. (2023)^18^	Prospective cohort study analysis	Severe MR due to myxomatous MV degeneration (NYHA I–II)	88 (40.9%)	55 [44–63] median [IQR]	88 (81/5/2 percutaneous repair)	Referred for surgical treatment of severe MR Pre‐ and postoperative 24 h Holter monitoring and TTE
Pandis, et al. (2024)^22^	Retrospective cohort analysis of progressively captured data	Elective first‐time cardiovascular MV repair for severe MR with diagnosed aMVP	62 (42%)	59.5 [51–65] median [IQR]	62 (62/0/0)	Preoperative TTE Baseline 12‐lead electrocardiograms (ECGs; *n* = 62/62) and ambulatory rhythm monitoring (*n* = 48/62) were analysed for the presence, morphology, and ectopic PVC burden. Postoperative follow‐up visits (up to 1 year after) including physical examination, imaging, 12‐lead ECG, Holter monitoring, and/or remote ambulatory telemetry report

*Note*: Age is presented as mean ± standard deviation or median and interquartile range [IQR].

Abbreviations: BiMVP, Bileaflet MVP; CRT, Cardiac Resynchronization Therapy; ECG, Electrocardiogram; HF, Heart failure; ICD, Internal Cardioverter Defibrillator / Implantable Cardiac Device; iMVP, isolated MVP; LVEF, Left Ventricular Ejection Fraction; MAD, Mitral Annular Disjunction; MR, Mitral Regurgitation; MV, Mitral Valve; MVP, Mitral Valve Prolapse; MVR, Mitral Valve Repair; MVS, Mitral Valve Surgery; NYHA, New York Heart Association; TOE, Transoesophageal Echocardiogram; TTE, Transthoracic Echocardiogram.

**TABLE 2:** Included cohort studies, study populations, length of follow‐up, and whether there was a reduction in VA or SCD post‐MVS.

References	Medical/surgical intervention	Study population	Follow‐up	Change in VA burden or SCD following intervention				
				PVC	NSVT	VT	VF	SCD
Reece, et al. (1985)^23^	33 underwent Mitral annuloplasty using a collar prosthesis 4 underwent annuloplasty using commissural plication	MVP with or without MR	Average follow‐up: 4.7 years (range 1–10) (175.6 patient‐years)	↓non‐VT/VF (proportion of PVC vs. NSVT not stated)		↓	↓	—
Grigioni, et al. (1999)^19^	Specific medical therapy not stated Specific surgical technique not stated	MR due to flail‐leaflet (and likely MVP based on exclusion criteria)	48 ± 41 months	—	—	—	—	↓
Olafiranye, et al. (2013)^21^	35 (61%) MV replacement 22 (39%) MV repair	Hemodynamically severe, nonischemic chronic MR (MVP in 75%)	Average follow‐up: 9.52 ± 3.49 years (range: 1.98–15.5) among endpoint‐free patients	—	↑	—	—	—
Naksuk, et al. (2016)^20^	30 (94%) MV repair 6 (18.8%) concomitant CABG	Symptomatic severe MR due to BiMVP	6.3 ± 5.3 years	↓ <60 years old	—	—	—	—
				↔ in overall cohort				
Vaidya, et al. (2016)^24^	Case 1: MV repair with prophylactic maze procedure Case 2 (Marfan): MVS Case 3: MV repair Case 4: MV repair and Bi‐atrial maze procedure with LA ligation Case 5 (hypertrophic cardiomyopathy and massive septal hypertrophy): MV repair, tricuspid valve repair, maze procedure, LA appendage closure and LA reduction atrioplasty	BiMVP and malignant arrhythmia with ICD	4.6 ± 2.9 years pre‐surgery 6.6 ± 4.2 years post‐surgery	↓	—	↓	↓	—
Essayagh, et al. (2021)^2^	170 (93%) MV repair 13 (7%) MV replacement Specific surgical technique not stated	iMVP with or without flail leaflet stratified by MAD (BiMVP in 47%)	10.3 ± 3.0 years	↓composite endpoint of VT, arrhythmia ablation of VT or disabling PVCs, cardioverter‐defibrillator implantation and SCD				
Ascione, et al. (2023)^18^	63 from 88 consecutive MVS patients meeting inclusion criteria All 63 patients underwent MV repair (followed by an annuloplasty with a posterior band [median ringsize 38 mm (37–39)])	Severe MR due to myxomatous MV degeneration (NYHA I–II)	24 h Holter monitoring prior to surgery 3 months of echocardiographic and Holter monitoring postintervention	↔ in overall cohort	↔ in overall cohort	—	↓	—
				↓ in those arrhythmogenic at baseline	↓ in those arrhythmogenic at baseline			
Pandis, et al. (2024)^22^	62 first‐time MV repairs	aMVP cases (characterised by degenerative mitral prolapse with frequent or complex VA (Lown grade ≥2)) with severe degenerative MR	Baseline 12‐lead ECGs and ambulatory rhythm monitoring 30 day and 1 year 12‐lead ECG, Holter monitoring, and/or remote ambulatory telemetry reports	↓minor‐VA = Lown grade 2 (frequent, isolated unifocal PVCs)				—
				↓ complex‐VA = Lown grade ≥3 (pleiomorphic PVCs, couplets/triplets, VT)				

*Note*: Green = Decrease in VA burden; Red = Increase in VA burden. Abbreviations: BiMVP, bileaflet MVP; ECG, electrocardiogram; HFrEF, heart failure reduced ejection fraction; ICD, internal cardioverter defibrillator/implantable cardiac device; LA, left atrium; LV, left ventricle; LVEF, LV ejection fraction; MAD, mitral annular disjunction; MR, mitral regurgitation; MVP, mitral valve prolapse; MVR, mitral valve repair; MVS, mitral valve surgery; NYHA, New York Heart Association; PVC, premature ventricular complex; SCD, sudden cardiac death; VA, ventricular arrhythmia; VF, ventricular fibrillation; VT, ventricular tachycardia.

#### 3.1.1 PVC burden pre‐ and post‐MVS

All three MVS cohort studies^18^, ^20^, ^24^ with rates of PVC pre‐ and postintervention reported a reduction in burden. Interestingly, Naksuk et al. in 2016^20^ found MVS did not uniformly reduce ventricular ectopy (VE) frequency in patients with Bileaflet MVP (BiMVP), finding instead a reduction only in those aged less than 60 (no significant reduction associated within the entire cohort with mean age of 63.5 ± 12.8 years). Each 10‐year decrease in age was associated with a reduction in VE frequency by 1.9 times [95% CI: 1.04–4.3 per 10‐year; *p* = 0.04] suggesting a graded relationship between age and odds of VA reduction with surgery.

Vaidya et al.^24^ reported in their 2016 retrospective analysis of five patients with BiMVP a reduction in PVC burden detected by implantable cardioverter‐defibrillator (ICD) interrogation in two of these patients (patient 1 PVC burden reduced from 1717 to 1184/24 h and patient 5 from 251 to 194/24 h).

Most recently, Ascione et al.^18^ in their 2023 prospective study investigating the impact of MVS on VA in 63 of their 88 consecutive patients with Barlow's disease (BD), using 24 h Holter monitoring prior to surgery and echocardiographic and Holter monitoring 3 months following their MV repair, reported almost half of the subjects arrhythmogenic at baseline became free from significant VA (defined as >1% PVC per 24 h period or at least one episode NSVT, VT or VF). However, they also observed in some cases an increase in PVC burden at follow‐up, demonstrated by an overall PVC >1% per 24 h period incidence rate of 9.3 (2.5–23.8) per 100 person‐years at risk in those patients classified non‐arrhythmogenic at baseline.

In this same study,^18^ PVC burden per 24 h increased from 3101 [996–8397.5] to 5661 [329–12143.5] in 9 patients persistently arrhythmogenic pre‐ and postintervention, decreasing from 1889 [266–3277] to 90 [20; 351] in 11 patients who became non‐arrhythmogenic. PVC burden per 24 h increased from 463 [29.2–699.2] to 765 [195.7–1602] in eight patients who became arrhythmogenic post‐MVS and increased from 8 [2–94] to 10 [2–31] in the remaining persistently non‐arrhythmogenic 35 patients.

#### 3.1.2 NSVT pre‐ and post‐MVS

Three MVS cohort studies reported rates of NSVT pre‐ and postintervention.^18^, ^21^, ^23^ Reece et al.'s^23^ 1985 prospective cohort studied 37 MVP patients with or without MR who underwent MV annuloplasty (33 collar prosthesis, four commissural plication), reporting a reduction in VA from 12 patients with non‐VT/VF VA to 0 patients (the proportion of PVC/NSVT not stated).

Olafiranye et al.'s 2013 study^21^ of 57 patients with non‐ischemic MR (MVP in 75%) who underwent MVS (22 MV repair and 35 MV replacement) reported the predisposition to arrhythmia persisted even after the abnormal mechanical effects of MR had been alleviated. Seventeen patients (33.3%) had NSVT prior to intervention (10 with >1 episode) compared to 19 patients (37.3%) within 18 months after MVS; nine were persistent with three greater than one episode, and ten were new cases. How soon these VAs were detected after surgery was not stated. The authors' interpretation of persistent arrhythmic risk, predicated on average annual risks of cardiovascular death and SCD post‐MVS, was based on the number of VT episodes (all nonsustained) albeit importantly was not compared to medical management. Average annual risk of cardiovascular death for 0 versus 1 versus >1 episodes of NSVT post‐MVS was 1.3% versus 2.8% versus 6.2% respectively. Annual risks for SCD were 0.6% versus 1.4% versus 4.9% respectively.

Ascione et al.^18^ (2023) reported the overall number of patients with NSVT reduced from 14 to 11 across 63 patients who underwent MV repair. Six patients developing new NSVT became arrhythmogenic following MV repair combined with five patients recording persistent NSVT. Seven of the 14 patients recorded episodes of NSVT at baseline but zero further episodes at 3‐month follow‐up.

#### 3.1.3 Sustained VA pre‐ and post‐MVS

Four MVS cohort studies reported a reduction in sustained VT or VF postintervention.^2^, ^18^, ^23^, ^24^ Two and three, respectively, of the 33 patients in Reece et al.'s^23^ 1985 cohort who experienced VF and VT, prior to MVS, recorded no episodes of VF or VT following MVS over an average follow‐up of 4.7 years (range 1–10 years, 175.6 patient‐years). The surgical method employed in these five patients was not stated.

Vaidya et al.^24^ reported a cumulative reduction in VA episodes post‐MVS detected by ICD interrogation as well as appropriate ICD therapies in all five patients with BiMVP and malignant arrhythmia (VF in all but one patient). There was an overall reduction across these five patients from 8 VT, 8 VF, and 4 VT/VF episodes to two episodes of VT and one episode of VF (6.0 ± 2.9 years pre‐surgery and 6.6 ± 4.2 years post‐surgery). Comparing pre‐ and post‐surgery, episodes of VF reduced from 0.6 to 0.14 events per person‐year, VT 0.4 to 0.05 events per person‐year as well as a reduction in ICD shocks from 0.95 to 0.19 events per person‐year.

Essayagh et al.'s 2021 analysis^2^ investigated the link between the presence of Mitral Annular Disjunction (MAD) at the time of MVP diagnosis and arrhythmic events, comparing medical to surgical intervention in controlling VA burden. Comprehensively characterized by TTE and 24 h‐Holter ECG, 170 of the 595 MVP patients had clinical arrhythmic events during a mean follow‐up of 10.3 ± 3.0 years (159 with VT ≥30 days post diagnosis, 14 VT or disabling PVC ablation, 14 ICD implantation, 3 SCD). Multivariate analysis showed an adjusted hazard ratio (HR) for severe VA events in the presence of MAD at MVP diagnosis of 3.21 [95% CI: 2.03–5.06; *p* < 0.0001] with medical management, compared with a time‐dependent surgery reduced HR of 2.54 [95% CI: 1.84–3.50; *p* < 0.0001].

VF was recorded during baseline monitoring in only two (a subset of the 11 patients who became non‐arrhythmogenic following MV repair) of the 63 patients undergoing MVS in Ascione et al.'s^18^ 2023 analysis, despite four patients having an ICD as a secondary prevention therapy (three patients with prior history of VF and one with history of symptomatic VT). No further episodes of VF were recorded post‐MV repair. The incidence rate of absence of significant VA (>1% PVC per 24 h period or at least one episode of NSVT, VT or VF) at follow‐up among patients arrhythmogenic at baseline was 55 (27.5–98.4) per 100 person‐years at risk (incidence rates of VT and VF alone not given). The incidence rate of significant arrhythmic burden at follow‐up among baseline non‐arrhythmogenic patients was 18.6 (8–36.7) per 100 person‐years at risk (NSVT, VT or VF incidence specifically being 14 (5.1–30.4) per 100 person‐years at risk).

Pandis et al.^22^ (2024) evaluated the cumulative incidence of VA at 1 year following surgical MV repair in patients with aMVP. Among 204 consecutive cases of severe degenerative MR undergoing first‐time MV repair between January 2018 and December 2020, 62 patients met the criteria for aMVP, characterized by degenerative mitral prolapse with frequent or complex VA (Lown grade ≥2). The cohort included 36 patients (58%) with complex VA (Lown grade ≥3) and 26 patients (42%) with minor VA (Lown grade = 2). At 1 year follow‐up (*n* = 54), freedom from recurrent VA was 75.9%, with significant differences based on preoperative VA severity. In the 24% of patients with recurrent VA (*n* = 13), almost all (*n* = 12 of 13, 92.3%) had pre‐existing complex VA or VT prior to surgery. Importantly, patients with minor VA experienced no postoperative recurrence. Complex VA was the strongest predictor of recurrence, increasing the risk by a factor of 10.8 [HR 10.8, 95% CI: 1.4–84.2; *p* = 0.024]. Additionally, patients with complex VA were more likely to undergo surgery at a younger age [55 versus 63.5 years; *p* = 0.029] and had a younger age of VA diagnosis [47 versus 57.4 years; *p* = 0.007].

#### 3.1.4 SCD: medical management versus MVS

Grigioni et al. in 1999^19^ were the only identified group to investigate and report a reduction in SCD following MVS (compared to medical management) despite similar patient characteristics in their MR with flail mitral leaflet (and likely MVP based on exclusion criteria) cohort. In 348 patients (14% NYHA III‐IV and 82% ≥ grade 3 MR) and a mean follow up of 48 ± 41 months, there were 99 deaths: 49 being cardiovascular death, 25 SCD in those with conservative management, and 7 SCD post‐MVS. Surgical correction of MR (due to flail‐leaflet and excluding papillary muscle rupture, previous valve surgery, moderate/severe MS or AV disease and congenital heart disease) performed irrespective of time from diagnosis was associated with a reduced SCD incidence (adjusted HR for SCD compared with medical management was 0.29 [0.11–0.72; *p* = 0.007]).

## 4 DISCUSSION

This manuscript seeks to highlight the evolving concept of the influence of MVS on VA risk and emphasize the importance of establishing a standardized framework for future studies evaluating its impact. The principal findings of this systematic review are:

1. Identified studies show a likely reduction in VA burden following MVS for aMVP (when surgery is performed in line with current guideline directed indications),
2. Residual VA and SCD risk remain despite surgical intervention, and
3. There is limited data as well as significant heterogeneity in the literature regarding trial design, patient cohorts, surgical technique, endpoint definitions, and reporting of outcomes.

### 4.1 Mitral valve surgery—current guidelines and its role in treating malignant MVP

Despite MVS for MR improving survival benefit irrespective of symptom status^8^, ^12^ or preoperative LVEF and/or RVEF,^33^ the current American (2020)^34^ and European (2021)^35^ surgical guidelines for valvular heart disease do not consider arrhythmia burden. Instead, algorithms are based on severity of MR, symptoms, LV dynamics, new‐onset AF, pulmonary artery pressure, and level of operability risk.

The recent EHRA consensus statement^36^ suggests the role of MVS for patients with aMVP remains controversial. While there is clear evidence for MVS in patients with symptomatic severe MR, and for those not eligible for surgery to instead receive optimal HF medication, whether successful mitral surgery provides sufficient treatment as a stand‐alone therapy to prevent VA in aMVP patients (in those with or without MR) remains unknown. How exactly MVS might reduce malignant arrhythmias in this population remains an accepted gap in knowledge, currently unable to be explained under a single unifying etiology.

Highlighting a reduction in VA following MVS, this systematic review of patients referred predominantly due to severe MR supports the mechanistic notion that adequate correction of MR and subsequent reduction of mechanical stress on the mitral leaflets, annulus, chordae, and papillary muscles, even in the presence of MAD, may help minimize the trigger for VA, albeit not eliminating risk completely.

Other mechanisms including stabilization of the mitral apparatus with annuloplasty, correction of MAD, and presence of fibrosis all likely determine VA risk postintervention and remain important considerations requiring future studies and better characterization.

It is important to acknowledge that the current body of evidence does not provide adequate support for MVS as a standalone arrhythmia‐specific intervention. In the studies reviewed, MVS was performed primarily for established guideline‐based indications, and any observed arrhythmic benefit remains for now a secondary and hypothesis‐generating observation. The potential for earlier intervention to interrupt proarrhythmic mechanisms in aMVP represents an exciting opportunity for future prospective investigation focused on arrhythmic outcomes.

The frequently reported reduction in VE or NSVT following surgery must be interpreted with caution, as these surrogate endpoints have not been conclusively linked to a reduction in SCD. The relationship between arrhythmia burden and true arrhythmic events is complex and often conflated. Notably, this study demonstrates that cases of VT/VF or SCA still occur despite surgery, suggesting residual risk persists in a subset of patients. The magnitude of any risk reduction remains uncertain, and the current findings do not allow for identification of those most likely to benefit following mitral intervention, or conversely, those who remain at risk of VA.

This underscores the need for more rigorous, prospective studies designed with sufficient granularity to disentangle these variables, assess meaningful clinical outcomes (such as sustained VT/VF or SCD), and ultimately identify which patients might derive the most benefit (or remain at residual risk) following mitral intervention. Addressing these limitations is critical to advancing our understanding of arrhythmia risk in aMVP and to inform any future shift in clinical management.

### 4.2 Mitral regurgitation and arrhythmogenic MVP

The 2022 EHRA consensus statement^36^ recognizes two key aMVP phenotypes: one associated with severe degenerative MR, where moderate to severe MR confers an increased risk of mortality and SCD; and another characterized by severe myxomatous MVP, in which VAs occur independently of MR severity, sex, or LV function. There is currently no literature specifically exploring whether MVS leads to a differential reduction in VAs or SCD when comparing patients with moderate versus severe mitral regurgitation.

The EHRA guideline's acknowledgement of an elevated SCD risk associated with severe myxomatous MVP phenotypes, irrespective of MR severity, is of novel importance in that it emphasizes the absence of hemodynamically significant MR should provide no particular reassurance in this population.^36^, ^37^ Highlighted in our 2018 systematic review^4^ of 57 SCD or cardiac arrest cases with isolated MVP (iMVP; whereby other potential causes of death are excluded) of which BiMVP was present in 40 (70%), importantly 83% of cases were associated with nonsevere MR.

MR is the second most common valvular disorder worldwide,^38^ with primary MR (due to intrinsic lesions of the MV apparatus) most commonly caused by a prolapsing degenerative MV (Carpentier type II classification). A 2018 prospective study of 356 patients with primary MR (177 with MVP diagnosed using Cardiac Magnetic Resonance (CMR)) and without other significant cardiac disease described a 1.2% annual event rate of SCD or malignant VA associated with MVP.^39^ Functional (secondary) MR occurs without organic MV disease, due instead to cardiac remodeling often in the context of advanced HF and thus associated with very high surgical risk. Moderate to severe MR in MVP and the sequelae of LV volume overload is a well‐accepted determinant of VA and SCD, and is prognostically significant for cardiac death secondary to HF, myocardial infarction and/or VA.^40^ Severe MR is present in approximately 7% of patients with MVP.^41^

Although surgical correction (or reduction) in MR and a pathologically elevated preload should in principle decrease congestive symptoms, myocardial wall stress, and improve hemodynamic performance, this review demonstrates that a residual risk of arrhythmia secondary to other postulated mechanisms linking MVP to VA and SCD remains.

### 4.3 Pro‐arrhythmic potential of valve surgery: distinguishing new‐onset versus persistent ventricular arrhythmia

The pro‐arrhythmic potential of MVS is recognized as an important yet complex phenomenon, and the occurrence of VA post‐surgery raises questions about the interplay between surgical intervention and underlying myocardial substrate. While corrective valve surgery such as MV repair can alleviate severe MR, it may also create a substrate conducive to arrhythmogenesis including scar‐related re‐entrant VA.^42^

Although sustained arrhythmic risk remains following MVR, reflecting persistent underlying myocardial substrate, there is emerging work investigating whether MV repair may be associated with reverse myocardial remodelling in some patients. Postoperative reductions in extracellular volume fraction (ECV%) and indexed extracellular volume (iECV), as measured by cardiac MRI, could reflect a decrease in diffuse myocardial fibrosis.^43^ However, these findings are indirect and limited by the absence of late gadolinium enhancement (LGE) change or histopathological confirmation. While surgical intervention may stabilize valve mechanics and hemodynamics, potentially modifying the arrhythmic milieu, the persistence of VA and SCD cases reported after surgery suggests ongoing residual risk and likely persistence of underlying substrate.

Illustrated by Ascione et al.'s^18^ 18.6% of non‐arrhythmogenic MVP patients developing an increased VA burden at 3‐month follow‐up despite successful MV repair (with complete abolition of MAD), it is plausible that alternative electrical foci not addressed by MV repair are responsible for VA. However, distinguishing these de novo arrhythmias from persistent pre‐surgical VAs driven by pre‐existing myocardial scar adds another layer of complexity.^28^

Pre‐existing LV scarring, prevalent in patients with severe MR, can sustain re‐entrant circuits independent of surgical intervention. These circuits may persist post‐surgery, complicating the attribution of arrhythmic events to surgical factors alone. Additionally, the role of non‐re‐entrant mechanisms, including arrhythmias linked to degenerative MVP and the natural progression of BD with increasing myocardial excitability, remains underexplored.^4^ Non‐re‐entrant VAs may emerge years after surgery, potentially driven by progressive myocardial changes, underscoring the heterogeneity of arrhythmic mechanisms in this population.

Another important consideration in the pro‐arrhythmic potential of valve surgery is whether the continued stretch of papillary muscles following repair, compared to replacement, influences postoperative arrhythmia burden. In repair techniques that preserve native chordal structures, residual mechanical stress on the papillary muscles—particularly in cases of incomplete leaflet coaptation or ongoing ventricular remodeling—may contribute to persistent arrhythmogenesis. The use of neo‐chordae in MV repair could also play a role, as the altered tension dynamics and potential for nonphysiologic stretch might influence ventricular electrophysiology.

Surprisingly, there is a significant lack of robust data and evidence regarding the incidence and mechanistic diversity of post‐MVS VA. While scar‐related re‐entry is likely predominant, the contribution of non‐re‐entrant mechanisms cannot be dismissed. Validation from prospective studies is necessary to clarify the role of surgical intervention in either mitigating or precipitating arrhythmic risk, particularly in patients with aMVP.

### 4.4 Timing of intervention

Data which suggests earlier surgical correction of MVP greater reduces VA burden has the potential to influence future guidelines concerning aMVP management. Studies such as Naksuk et al.'s^20^ which demonstrate VA reduction predominantly in individuals under 60 years of age with BiMVP, reveal a graded relationship between age and odds of VA reduction with surgery. Age as an independent variable in VA reduction supports the hypothesis that a progressive arrhythmic substrate develops over time in the context of longstanding MVP. Suppressing the progression of valve prolapse and its sequelae may play a crucial role in preventing VA/SCD, further substantiating the mechanical theory of VA in aMVP and the potential benefit of early MVS in altering electrophysiologic substrate.

Similarly, Pandis et al.^22^ further demonstrate that intervention before the progression of VA to complex forms may significantly reduce postoperative VA burden. Complex baseline VA emerged as the strongest independent predictor of recurrent VA in a cohort of 62 aMVP patients. Additionally, the high prevalence of family history of MVP among survivors of SCA in their cohort reinforces the importance of integrating risk stratification tools to identify high‐risk patients early and potentially tailor surgical interventions to maximise clinical benefit.

In the studies reviewed, MVS was undertaken for established guideline‐based indications, primarily in the context of hemodynamically significant MR. While these findings are insufficient to change current guideline‐based indications, they nonetheless offer hypothesis‐generating insights that mitral valve intervention may alter the arrhythmic risk in an MVP patient.

### 4.5 Mitral annular disjunction

VA occurrence correlates with the severity of MAD^36^; an abnormal atrial displacement of the MV leaflet hinge point observed only at the insertion of the posterior mitral leaflet (limited anteriorly by the mitro‐aortic fibrous continuity and therefore not involving the anterior leaflet), causing systolic separation between the ventricular myocardium and the mitral annulus, with upper and lower limits measured (CMR or echocardiography in the parasternal long‐axis view during end‐systole) between the level of posterior leaflet insertion on the annulus/left‐atrial‐wall and the level of the LV myocardium, respectively.

Adjusting for age, sex, Charlson index, symptoms, AF, LVEF, and MR grade, Essayagh et al.'s (2021)^2^ reported a persistent link between MAD and arrhythmic events when accounting for the timing of operation after diagnosis. A weaker association compared to medical management suggests some benefit from MVS in reducing MAD‐related VA burden.

The annular structural integrity and potential disappearance of MAD achieved by MVS due to the suturing of the ring and prosthesis and joining of the annulus to the LV myocardium^44^, ^45^ is hypothesized to reduce VA burden further. Whether additive therapy for MAD and stabilization of the mitral annulus is necessary requires clarification.^46^

### 4.6 Sudden cardiac death

An important consideration regarding Grigioni et al.'s^19^ findings of reduced SCD is no data being presented concerning the incidence of VA pre‐ and post‐MVS; a source of ambiguity regarding whether such reduction can be attributed to correction of aMVP or instead a function of other entities including HF. In support of the former, despite NYHA functional class and LVEF being reported as independent predictors of SCD under medical management in multivariate analysis, 20% of their 25 conservatively managed SCD patients had no evidence of AF, LV dysfunction, nor severe symptoms (15 of the 19 SCD patients in functional class I‐II had LVEF ≥60%). Similarly, in 10 of these patients where cardiac rhythm was ascertained, VT or VF occurred despite only seven individuals having a possible history of CAD.

### 4.7 Predictors of VA or SCD pre‐ and post‐MVS

Table 3 presents each cohort study's reported heterogeneous group of clinical, electrical, and imaging predictors associated with various outcomes including VA or SCD.

**TABLE 3:** Included cohort studies' predictors of reduction in VA or SCD incidence following intervention, and other measured endpoints.

References	Outcomes/endpoints reported in each cohort study	Predictor/association
Reece, et al. (1985)^23^	Reduction in VA burden	None specified
	Improvement in NYHA functional class	Functional improvement more uniform in patients with associated MR
	Chest pain	None specified
	Palpitations	None specified
Grigioni, et al. (1999)^19^	SCD incidence	NYHA functional class, LVEF, AF, presence of severe symptoms
	SCD reduction post‐MVS	Surgical correction of MR at anytime
Olafiranye, et al. (2013)^21^	Prognostic value (mortality) of VT post‐MVS (modulated by LVEF and RVEF)	>1 VT episode within 18 months after MVS predicted late postoperative SCD and cardiovascular death Subnormal postoperative RVEF strong predictor for cardiovascular death but not SCD Normal RVEF and VT >1 episode predicted SCD
Naksuk, et al. (2016)^20^	PVC reduction post‐MVS	Age <60 Each 10‐year decrease in age was associated with a possibility of reduction in VE by 1.9
Vaidya, et al. (2016)^24^	Reduction in VA burden	None specified
Essayagh, et al. (2021)^2^	Overall survival (adjusted for age, sex, Charlson index, symptoms, AF, LVEF, and MR grade)	None identified when stratified by presence of MAD
	Freedom from clinical arrhythmic events (VT, VA ablation, ICD implantation, SCD)	MAD at time of MVP diagnosis (excess occurrence of events) MVS (compared to medical management)
	Presence of MAD	MVP, severe myxomatous disease involving BiMVP, leaflet redundancy, LV size (larger) Age (younger), Charlson index (lower), CAD (less), HF, HTN, AF, syncope, palpitations
Ascione, et al. (2023)^18^	Differences between arrhythmogenic and non‐arrhythmogenic Barlow's patients	Female, history of palpitations
	Reduction in VA burden	Infero‐lateral MAD
Pandis, et al. (2024)^22^	Recurrent VA at 1 year after mitral repair surgery	HTN, preoperative VA complex (Lown ≥3), VA‐related syncope, history of sudden death, QRS range (ms), inverted/biphasic T‐waves, conduction delay (LV or AV), fascicular block

Abbreviations: AF, atrial fibrillation; BiMVP, bileaflet MVP; CAD, coronary artery disease; HF, heart failure; HTN, hypertension; ICD, internal cardioverter defibrillator/implantable cardiac device; LV, left ventricle; LVEF, LV ejection fraction; MAD, mitral annular disjunction; MR, mitral regurgitation; MVP, mitral valve prolapse; MVS, mitral valve surgery; NYHA, New York Heart Association; PVC, premature ventricular complex; RVEF, right ventricular ejection fraction; SCD, sudden cardiac death; VA, ventricular arrhythmia; VE, ventricular ectopic; VF, ventricular fibrillation; VT, ventricular tachycardia.

Of note, Essayagh et al.'s 2021 analysis^2^ reported the presence of MAD at the time of MVP diagnosis to be independently associated with an excess occurrence of their clinically severe arrhythmic event composite endpoint: VT, arrhythmia ablation of VT or disabling PVCs, cardioverter‐defibrillator implantation, and SCD [HR: 2.16 (1.59–2.92); *p* < 0.0001]. However, MAD was not associated with excess mortality during the 10‐year follow‐up.

In this same cohort of 595 patients with iMVP (defined by the authors as MVP without valvular heart disease/intervention or congenital, infiltrative or arrhythmogenic cardiomyopathies), Essayagh et al.'s earlier 2020 prospective analysis^47^ reported severe VA to be independently associated with the presence of MAD, leaflet redundancy and repolarization abnormalities, but interestingly not with MR severity or ejection fraction. MAD was associated with a 7 times (odds ratio 6.97) more likely occurrence of severe VA and 3‐times (odds ratio 3.27) more likely occurrence of mild/moderate VA. The presence of T‐wave inversion or ST‐segment depression was associated with a twofold (odds ratio 2.30) increased risk of mild–moderate VA, with severe VA having an eightfold increased risk (odds ratio 8.04) and being independently associated with excess mortality. Overall mortality after arrhythmia diagnosis was strongly associated with arrhythmia severity. It should be noted however the not insignificant proportion of patients with CAD (23%) and severe MR (28%) in this cohort, both also independently associated with VA, perhaps warranting caution with the term ‘isolated’ MVP.^6^

Ascione et al.^18^ reporting their MVP patients arrhythmogenic at baseline being more often females [*p* = 0.056] with a history of palpitations [*p* = 0.007] is in keeping with the well accepted classical aMVP phenotype. No other significant preoperative, intraoperative, or postoperative characteristics were found.

Similar to Essayagh's^45^ reported MAD prevalence of 44% among their 61 patients with MVP and severe MR, 35% of Ascione's^18^ MVP cohort displayed MAD. Despite being infero‐lateral in all cases, MAD prevalence preoperatively was no different between arrhythmogenic and nonarrhythmogenic groups, a finding contrary to Essayagh's and other large retrospective series.^2^, ^48^ Importantly, however, a higher prevalence of preoperative inferolateral MAD was reported in those with VA reduction post‐MVS compared to patients who remained arrhythmogenic [63.6% vs. 11.1%; *p* = 0.028]. These conflicting findings might perhaps in part be explained by the recent concept of true‐ and pseudo‐MAD.^49^ With most available studies defining MAD as a purely systolic phenomenon, the prevalence of pseudo‐MAD is likely underreported; true‐MAD prevalence perhaps being overestimated using echocardiographic imaging.

### 4.8 Surgical cryoablation

Vohra et al.^50^ recently (2022) reported three malignant MVP patients (refractory to medical therapy) who underwent surgical cryoablation at the time of MVS and remained free of VA during follow‐up, with no detrimental effect on valve function secondary to the cryoablation lesions. There is still, however, very limited evidence from case reports^50^, ^51^ regarding the efficacy of surgical cryoablation during MVS in patients with a history of complex VA, with long‐term outcomes and recommendations not yet defined.

## 5 IMPACT ON DAILY PRACTICE—DOES AMVP WARRANT EARLIER MITRAL INTERVENTION?

There is to date a lack of evidence‐based guidelines regarding the early identification and treatment of aMVP. We provide an integration of available data regarding whether MVS reduces the incidence of VA and SCD due to MVP. Important evidence gaps remain, including optimal timing of operation (ideally prior to malignant substrate formation), efficacy of technique employed (repair, replacement, annuloplasty, cryoablation, surgical, percutaneous, etc.), significance of varying degrees of MR pre‐ and postintervention, and whether stabilization of the MV apparatus or annulus decreases subsequent risk of VA or SCD.

While overall there seems to be a decrease in VA burden following surgical intervention, there remain VA and SCD post‐MVS and therefore risk in this aMVP cohort is not completely eliminated by surgery. Additional anti‐arrhythmic treatment, be it pharmacological or device‐based therapy, is likely beneficial in select patients and therefore further motivation to define quantifiable risk factors.

AMVP may warrant earlier mitral intervention in high‐risk patients due to the unpredictable and potentially early onset of complex VA (including VT and SCA). While some patients exhibit a slow progression of VA burden, others develop severe arrhythmic complications as early as the second decade of life, even when MR is not severe.

Further knowledge of malignant VA and SCD associated with MVP and other entities including MAD, either treated or untreated, is important given the potential of life‐saving ICD therapies to exist. The risks and complications of ICD insertion combined with the potential for long‐term psychological impacts on patients, as well as the high cost on health resources, make it essential that the choice of patient is done appropriately using the best available evidence.

### 5.1 Future studies and standardization of data

VA and SCD risk stratification in this cohort remains a significant challenge, and optimal treatment has not yet been achieved. This presents a unique opportunity to establish clear standards for the data elements and reporting practices necessary to meaningfully assess the impact of mitral surgery on VA risk. Consistent reporting of patient selection criteria, severity and mechanism of MR, presence and morphology of MVP and MAD, surgical details including repair versus replacement, along with systematic documentation of arrhythmic endpoints such as PVC burden, NSVT, sustained VT/VF, and SCA/SCD events is essential.

Uniform arrhythmia definitions, clear reporting of persistent versus new arrhythmia postintervention, a standardized follow‐up duration with regular interval 12‐lead ECG and Holter monitoring, combined with multimodality imaging including echocardiogram, exercise stress testing, and CMR imaging, pre‐ and postintervention, will likely more clearly define in whom and at what symptom point interventions such as MVS for arrhythmic burden should be considered, and whether an ICD should be utilized for the primary prevention of SCD in this high‐risk cohort.

Clinical trials are necessary albeit the rare occurrence of events, with the currently unmet need for and the potential benefit of a validated risk stratification system obvious. Instead, future cohort studies incorporating standardised and well‐defined endpoints, with clear definitions and sufficient reporting of data, would allow meta‐analyses to further our knowledge in this area. Development of a risk stratification scoring system would benefit from multicenter collaborative data collection using a central registry.

## 6 LIMITATIONS

The main limitation of this systematic review is a paucity of data addressing this issue; with substantial heterogeneity regarding trial design, measured outcomes, reporting standards, and patient cohorts existing within the small number of published studies. Included studies often measured only certain VA parameters, rarely reported whether VA events were new or persistent or how soon these occurred postintervention, definitions of arrhythmia were not uniform, and specifics of surgical technique employed were lacking. Further, there were major differences regarding follow‐up duration before and after surgery between studies.

Available studies ranged from 1985 to 2024 (with improved treatment modalities over time), with patient cohorts varying substantially regarding history of MR, presence of MAD, and range of LVEF. Similarly, anti‐arrhythmic regimes varied between studies, with baseline intra‐study dosing before surgery also often varying with postsurgery dosing. The retrospective nature of the available studies introduces substantial heterogeneity, and limitations in study design hinder the ability to isolate the mechanistic drivers of arrhythmic improvement, whether due to ventricular unloading, annular stabilization, or other structural and electrophysiological changes.

The studies included in this review are also affected by biases in patient selection, referral processes, and treatment allocation. Differences in the origin and severity of VA and the associated proarrhythmic substrate introduce challenges in comparing and achieving consistent outcomes following surgical correction of aMVP. Moreover, disparities in methods used both to identify aMVP or VA, or to determine the threshold/indication for surgical repair, introduce variations in referral practices and treatment timing, further complicating the evaluation of mitral repair's role in reducing VA‐related risks. Patients undergoing earlier interventions may experience distinct arrhythmic and MR risk profiles compared to those treated later. A tabulated summary of the risk of bias assessment, conducted using the ROBINS‐I (Risk Of Bias In Nonrandomized Studies—of Interventions) tool and focused specifically on the effect of mitral intervention on arrhythmic outcomes in MVP, is provided in Table S7.

While acknowledged limitations, these do highlight the need for a standardized reporting approach in these surgical and interventional cohorts and multicenter collaborative studies. Until this is achieved, the possible mechanisms by which MVS might reduce VA burden remain purely speculative.

## 7 CONCLUSIONS

This systematic review highlights a likely reduction in VA burden following MVS for aMVP. However, a residual risk of VA and SCD remains. Guidelines for surgical correction of MVP based on arrhythmic and SCD risk are lacking, and the underlying mechanisms for VA and SCD in these cohorts remain incompletely understood. Further development of risk stratification strategies to identify patients who may benefit from MV intervention and those who have a residual risk of VA and SCD is warranted.

## FUNDING INFORMATION

Dr. Lim has received funding from the Australian Government's Medical Research Future Fund; travel expenses from the Asia Pacific Heart Rhythm Society Conference; and research support from Abbott and Medtronic, and is the Australian Representative for the Asia Pacific Heart Rhythm Society. Dr. Mahajan has served on the advisory board of Abbott and Medtronic. The University of Adelaide reports receiving on behalf of Dr. Mahajan lecture and/or consulting fees from Abbott, Bayer, Biotronik, Medtronic, and Pfizer. The University of Adelaide reports receiving on behalf of Dr. Mahajan research funding from Abbott, Bayer, and Medtronic. Dr. Sanders has received funding from Boston Scientific, Medtronic, Abbott Medical, and Becton Dickinson, speaker fees from Pfizer, and a fellowship from the National Health and Medical Research Council of Australia; and has served on the advisory boards of Medtronic, Abbott Medical, Boston Scientific, CathRx, and Pacemate. The other authors have no relevant disclosures to report.

## CONFLICT OF INTEREST STATEMENT

The authors declare no conflicts of interest.

## Supporting information


Data S1.

## Data Availability

The data and study materials will not be made available to other researchers for purposes of reproducing the results or replicating the procedure, as source data for this systematic review are available from web‐based medical libraries.
