# Supplementary material for: Arrhythmogenic mitral valve prolapse—a systematic review of ventricular arrhythmia and sudden cardiac death outcomes before and after mitral valve surgery
Source: J Arrhythm. 2025 Jul 15;41(4):e70108. doi: 10.1002/joa3.70108 (PMC12264319; doi:10.1002/joa3.70108)
Supplement: Supplementary file 1 — Data S1. [file JOA3-41-e70108-s001.docx]

**Arrhythmogenic Mitral Valve Prolapse – A Systematic Review of Ventricular Arrhythmia and Sudden Cardiac Death Outcomes Before and After Mitral Valve Surgery**

**SUPPLEMENTAL MATERIAL**

James N. Cameron BSc MEng MBBS^1,2^, Nigel Sutherland BPhysio MD FRACP^3^, Chee Loong Chow MBBS FRACP^2,3^, Hui-Chen Han MBBS PhD FRACP^4^, Matias Yudi MBBS PhD FRACP^1,2^, Rajiv Mahajan MBBS MD PhD FRACP^5,6^, Anand Ganesan MBBS PhD FRACP^7^, Avi Sabbag MD PhD^8^, Kristina H. Haugaa^9^, Jai Raman MBBS PhD FRACS^10^, Prashanthan Sanders MBBS PhD FRACP^11^, Omar Farouque MBBS PhD FRACP^1,2^, Han S. Lim MBBS PhD FRACP^1,2#^

1. Department of Cardiology, Austin Health, Melbourne, Australia
2. Faculty of Medicine, Dentistry and Health Sciences, University of Melbourne, Melbourne, Australia
3. Department of Cardiology, Northern Health, Melbourne, Australia
4. Victorian Heart Institute, Monash University, Clayton, VIC, Australia
5. Adelaide Medical School, University of Adelaide, Adelaide, Australia
6. Lyell McEwin Hospital, Northern Adelaide Health Local Health Network, Elizabeth Vale, South Australia, Australia
7. Department of Cardiovascular Medicine, Flinders Medical Centre, Adelaide, South Australia, Australia
8. The Davidai Center for Rhythm Disturbances and Pacing, Chaim Sheba Medical Center, Tel Hashomer 52621, Israel
9. ProCardio Center for Cardiological Innovation, Department of Cardiology, Oslo University Hospital, Rikshospitalet, Sognsvannsveien 20, Oslo 0372, Norway and University of Oslo, Oslo, Norway
10. Department of Cardiac Surgery, Austin Health, Melbourne, Victoria
11. Centre for Heart Rhythm Disorders, South Australian Health and Medical Research Institute, University of Adelaide and Royal Adelaide Hospital, Adelaide, South Australia, Australia

# Corresponding Author

Associate Professor Han Lim

Email: [Lim.h@unimelb.edu.au](mailto:Lim.h@unimelb.edu.au)

Correspondence to: Department of Cardiology, Austin Health, Level 5N, 145 Studley Road, Heidelberg 3084, Victoria, Australia

Supplemental Table S1: Inclusion and exclusion criteria in included studies

| Reference | Inclusion Criteria | Exclusion Criteria |
| --- | --- | --- |
|  | **Cohort Studies** | |
|  | **MVS (repair / replacement)** | |
| [Reece, et al. (1985) ^1^](#_ENREF_1) | 37 symptomatic patients who underwent MVS between 1974 and 1983, with MVP, normal coronary arteries and no other cardiac pathology except that related to the Mitral valve | NA |
| [Grigioni, et al. (1999) ^2^](#_ENREF_2) | MR with flail leaflet diagnosed echocardiographically between 1980 and 1994 at Mayo Clinic | Surgical correction within 1 month diagnosis of MR-FL  Papillary muscle rupture  Previous valve surgery  Associated moderate or severe MS or AV disease  Congenital heart disease |
| [Olafiranye, et al. (2013) ^3^](#_ENREF_3) | MVS for hemodynamically severe, non-ischemic, pure, isolated chronic MR (verified by catheterization)  24-hour ambulatory electrocardiography (AECG) within 18 months after MVS and ongoing during 10-year follow up | Clinically evident coronary artery disease  >mild additional valve disease  Other structural heart disease |
| [Naksuk, et al. (2016) ^4^](#_ENREF_4) | Patients > 18 years-old undergoing MV surgery for MR secondary to bileaflet MVP at Mayo Clinic (2007-2013) who had available pre- and post-operative Holter monitoring data. | Those without pre- and post-operative Holter monitors  Patients without research authorization |
| [Vaidya, et al. (2016) ^5^](#_ENREF_5) | Underwent MVS from 1993-2013 at the Mayo Clinic  Bileaflet mitral valve prolapse and malignant arrhythmia with ICD in place both pre- and post-surgery  Pre-operative VT/VF | NA |
| [Essayagh, et al. (2021) ^6^](#_ENREF_6) | >= 18 years old, isolated MVP, with or without flail leaflet  First diagnosed at the Mayo Clinic in Rochester, Minnesota, from 2003 to 2011  Comprehensive clinical and echocardiographic evaluation at diagnosis, including symptoms, clinical history, and comorbidities  Arrhythmia evaluation by 24-h Holter monitoring during follow-up  Available electronic echocardiographic images for detailed morphologic assessment | Denied research-authorization  ≥moderate aortic regurgitation or stenosis, ≥moderate mitral stenosis or previous valvular surgery  Congenital heart disease (patent-foramen-ovale not excluded)  Hypertrophic, infiltrative, restrictive cardiomyopathy or pericardial restriction  Arrhythmic cardiomyopathy |
| [Ascione, et al. (2023) ^7^](#_ENREF_7) | From February 2021 to May 2022, referred for surgical treatment of severe MR  Barlow’s Disease  Successful follow-up protocol with  the first re-evaluation at 3 months | Not yet reaching the scheduled re-evaluation time point post-intervention  MV replacement or transcatheter MV repair |
| [Pandis, et al. (2024) ^8^](#_ENREF_8) | Elective MV repair for significant degenerative mitral regurgitation with diagnosed aMVP as a first-time cardiovascular intervention in a quaternary reference centre between January 2018 and December 2020  aMVP was defined as degenerative mitral prolapse with frequent or complex-VA (Lown-Wolf grade ≥2) | Non-aMVP (none or rare (<1/min or 30/h) isolated, monomorphic PVCs (Lown grade<2)) |
|  | **Case studies** | |
| [Ross, et al. (1978) ^9^](#_ENREF_9) | 35-year-old woman with proven MVP  Cardiac arrest in outpatient clinic (subsequent recording of serious ventricular tachyarrhythmias, deemed a likely episode of ventricular tachycardia or possibly ventricular fibrillation) | NA |
| [Pocock, et al. (1991) ^10^](#_ENREF_10) | 22-year-old nurse, 1966, with substernal chest pain (atypical) and palpitations. 3/6 late-onset systolic murmur  Ecg: small q waves and inferior TWI  Moderate billowing of the posterior leaflet and mild MR on Left Ventriculogram  Persistence of potentially fatal arrhythmias despite medical treatment | NA |
| [Bortnik, et al. (2009) ^11^](#_ENREF_11) | 80-year-old woman successfully resuscitated and referred to surgery after a cardiac arrest due to VF | NA |
| [Abbadi, et al. (2014) ^12^](#_ENREF_12) | 36-year-old Caucasian female with history of a benign murmur since adolescence  Sudden onset palpitations and dizziness in her 4th postpartum month, subsequently suffering a cardiac arrest  Echocardiogram showing bileaflet MVP with moderate mitral regurgitation and normal LV systolic function | NA |
| [Hosseini, et al. (2016) ^13^](#_ENREF_13) | Patient 1: 35-year-old Caucasian woman with a history of hypertension, syncope, chest pain, palpitations and dyspnoea.  Ecg: sinus rhythm and frequent premature ventricular contractions (PVCs) associated with a right bundle branch block, inferior axis morphology and wide QRS complexes, nonsustained ventricular tachycardia  Patient 2: 67-year-old Caucasian woman with a history of moderate-to-severe degenerative MR, frequent palpitations, an episode of aborted sudden cardiac death, and near-syncope episodes while on metoprolol 200 mg per day  Ecg: Frequent PVCs with a right bundle branch block, superior axis and a QRS width > 160 ms, episodes of nonsustained ventricular tachycardia | NA |
| [Augello, et al. (2017) ^14^](#_ENREF_14) | 46-year-old female with Barlow’s disease (MVP diagnosed aged 20)  Systolic curling of posterior LV wall and significant mitral annular disjunction  Complex ventricular arrhythmias | NA |
| [Alqarawi, et al. (2018) ^15^](#_ENREF_15) | 39-year-old female with severe MR secondary to MVP  Myxomatous mitral valve disease with bileaflet prolapse, normal LV size and ejection fraction, and normal pulmonary artery systolic pressure | NA |
| [Barman, et al. (2021) ^16^](#_ENREF_16) | 42-year-old female with palpitations and syncope  Diagnosis of MVP prior to surgery  Pre-surgery: baseline ECG showed T negativity in the inferolateral leads (II, III, AVF, V4-6)  Pre-surgery: ECG Holter monitoring revealed nonsustained VT. Ventricular premature beats were also observed with multifocal right bundle branch block morphology (suggesting LV origin) | NA |

Supplemental Table S2: Surgical method per patient per study

| Reference | Surgical method not documented | MV repair | MV replacement | MV repair along with maze procedure | Percutaneous MV repair with ‘MitraClip’ | MV replacement with mitral leaflets and/or papillary muscles excision | MV annuloplasty +/- valve repair | Annuloplasty with commissural plication |
| --- | --- | --- | --- | --- | --- | --- | --- | --- |
|  | **Cohort Studies** | | | | | | | |
|  | **MVS (repair / replacement)** | | | | | | | |
| [Reece, et al. (1985) ^1^](#_ENREF_1) |  |  |  |  |  |  | 33 (collar prosthesis, 13 rigid, 20 flexible) | 4 |
| [Grigioni, et al. (1999) ^2^](#_ENREF_2) | 186 |  |  |  |  |  |  |  |
| [Olafiranye, et al. (2013) ^3^](#_ENREF_3) |  | 22 | 35 |  |  |  |  |  |
| [Naksuk, et al. (2016) ^4^](#_ENREF_4) | 2 | 30 |  |  |  |  |  |  |
| [Vaidya, et al. (2016) ^5^](#_ENREF_5) |  | 3 | 1 | 1 |  |  |  |  |
| [Essayagh, et al. (2021) ^6^](#_ENREF_6) |  | 170 | 13 |  |  |  |  |  |
| [Ascione, et al. (2023) ^7^](#_ENREF_7) |  | 81 | 5 |  |  |  |  |  |
| [Pandis, et al. (2024) ^8^](#_ENREF_8) |  | 62 | 0 |  |  |  |  |  |
|  | **Case studies** | | | | | | | |
| [Ross, et al. (1978) ^9^](#_ENREF_9) |  |  |  |  |  | 1 |  |  |
| [Pocock, et al. (1991) ^10^](#_ENREF_10) |  |  |  |  |  |  | 1 |  |
| [Bortnik, et al. (2009) ^11^](#_ENREF_11) |  |  |  |  |  |  | 1 |  |
| [Abbadi, et al. (2014) ^12^](#_ENREF_12) |  |  |  |  |  |  | 1 |  |
| [Hosseini, et al. (2016) ^13^](#_ENREF_13) |  |  |  |  |  |  | 2 |  |
| [Augello, et al. (2017) ^14^](#_ENREF_14) |  |  |  |  |  |  | 1 |  |
| [Alqarawi, et al. (2018) ^15^](#_ENREF_15) |  |  |  | 1 |  |  |  |  |
| [Barman, et al. (2021) ^16^](#_ENREF_16) |  |  |  |  |  |  | 1 |  |

Supplemental Table S3: Definitions provided per study for Complex VA / SCD / Cardiovascular Death

| Reference | PVC | NSVT | Complex VA | Cardiac Death | Sudden Cardiac Death |
| --- | --- | --- | --- | --- | --- |
|  | **Cohort studies** | | | | |
|  | **MVS (repair / replacement)** | | | | |
| [Reece, et al. (1985) ^1^](#_ENREF_1) | - | - | - | - | - |
| [Grigioni, et al. (1999) ^2^](#_ENREF_2) | - | - | - | - | Occurred within 1 hour in a patient who was well or medically stable |
| [Olafiranye, et al. (2013) ^3^](#_ENREF_3) | - | ≥3 consecutive ventricular complexes; classified as non-sustained if total run was < 30 sec | - | Death resulting from heart failure (HF), determined by primary physician and cardiologist, in addition to all SD | Either witnessed unexpected arrest (resuscitated or non-resuscitated) in a patient who was previously stable or an unwitnessed unexpected death in a patient seen and apparently stable within the previous 24 hours |
| [Naksuk, et al. (2016) ^4^](#_ENREF_4) | - | - | - | - | - |
| [Vaidya, et al. (2016) ^5^](#_ENREF_5) | - | - | - | - | - |
| [Essayagh, et al. (2021) ^6^](#_ENREF_6) | - | - | clinical arrhythmic events (VT, arrhythmia ablation, cardioverter-defibrillator implantation, SCD)  Arrhythmia ablation = VT or disabling PVCs  VT: runs ≥3 beats with a rate ≥120 beats/min | - | - |
| [Ascione, et al. (2023) ^7^](#_ENREF_7) | - | - | - | - | - |
|  | Significant arrhythmic burden was defined as >1% PVB/24-hr period or at least one episode of NSVT, VT  or VF | | | | |
| [Pandis, et al. (2024) ^8^](#_ENREF_8) | VA was stratified as minor (m-VA) or complex (c-VA) according to the Lown-Wolf classification.  c-VA = Lown grade ≥ 3 (ie, pleiomorphic PVCs, couplets/triplets, ventricular tachycardia [VT]) and m-VA = Lown grade 2 (ie, frequent, isolated unifocal PVCs).  Patients with none or rare (<1/min or 30/h) isolated, monomorphic PVCs (Lown grade < 2) were classified as non-VA and excluded.  aMVP was defined as degenerative mitral prolapse with frequent or c-VA (grade ≥ 2) | | | | |

Supplemental Table S4: Endpoints - Each cohort study’s available quantitative data regarding rates (or risk reduction) of PVC burden, NSVT, complex VA, Cardiac Death (CD) and/or SCD pre- and post-intervention

| **Reference** | **PVC** | | **NSVT** | | **Complex VA** | | **Cardiac Death (CD)** | | **Sudden Cardiac Death (SCD)** | |
| --- | --- | --- | --- | --- | --- | --- | --- | --- | --- | --- |
|  | Pre-intervention | Post-intervention | Pre-intervention | Post-intervention | Pre-intervention | Post-intervention | Medical management | Post-intervention | Medical management | Post-intervention |
| [Reece, et al. (1985) ^1^](#_ENREF_1) | - | - | 17 total (unclear what proportion were PVC/NSVT)  15 ‘ventricular arrhythmias’  2 ‘ventricular and atrial arrhythmias’  2 were VF and 3 VT | 13 post-operative palpitations, 5 of which were ‘atrial arrythmias’ | 2 patients VF  3 patients VT | 0 VF  0 VT | - | 0 | - | 0 |
| [Grigioni, et al. (1999) ^2^](#_ENREF_2) | - | - | - | - | - | - | - | - | 25 sudden deaths | Surgery performed at any time:  Adjusted hazard ratio 0.29 [95% CI [0.11 to 0.72], p=0.007  7 sudden deaths |
| [Olafiranye, et al. (2013) ^3^](#_ENREF_3) | - | - | 33.3% cohort  (n = 17)  (10 with > 1 NSVT episode) | 37.3% cohort  (n=19)  9 persistent (3 with > 1 NSVT episode)  10 new | - | 0 | - | 0 vs 1 vs >1 NSVT after MVS:  Average annual risk 1.3% vs 2.8% vs 6.2% | - | 0 vs 1 vs >1 NSVT after MVS:  Average annual risk 0.6% vs 1.4% vs 4.9% |
| [Naksuk, et al. (2016) ^4^](#_ENREF_4) | Median 41 bph, IQR [16, 196] | Overall, the VE frequency was unchanged with surgery:  Median 40 bph, IQR [5, 186]; p = 0.34)  -13% median change, IQR [-77, 54]  Age and odds of VE reduction with surgery:  (odds ratio 1.9; 95% CI 1.04–4.3 per 10-year; p = 0.04)  Age <60 years:  reduction in postoperative VE (odds ratio 5.8; 95% CI, 1.1–44.7; p = 0.03) | - | - | - | - | - | - | - | - |
| [Vaidya, et al. (2016) ^5^](#_ENREF_5) | Patient 1: 1717/24hr  Patient 5: 251/24hr | Patient 1: 1184/24hr  Patient 5: 194/24hr | - | - | Across all 5 patients: | | - | - | - | - |
|  |  |  |  |  | 8 episodes VT  4 episodes (VT/VF)  8 episodes VF  Event rates per person-years:  VT 0.6  VF 0.4  ICD Shock 0.95 | 2 episodes VT  1 episode VF  Event rates per person-years:  VT 0.14  VF 0.05  IDC Shock 0.19 |  |  |  |  |
| Essayagh, et al. (2021) | - | - | - | - | 170 patients had clinical arrhythmic events follow-up: 159 VT≥30 days post diagnosis, 14 VT or disabling PVC ablation, 14 ICD implantation | | - | - | 3 from 170 patients’ clinical arrhythmic events were SCD |  |
|  |  |  |  |  | Link between presence of MAD (pre-surgery) and arrhythmic events in isolated MVP:  (Any severe VA event, Multivariate Analysis) | |  |  |  |  |
|  |  |  |  |  | Overall cohort: | |  |  |  |  |
|  |  |  |  |  | Medical management adjusted HR: 3.21; 95% CI: 2.03-5.06; p < 0.0001  Mitral surgery adjusted HR: 2.07; 95% CI: 1.24-3.43; p = 0.005 | Time-dependent surgery adjusted HR: 2.54; 95% CI: 1.84-3.50; p < 0.0001  VT risk after surgery did not reach significance - adjusted HR: 1.49; 95% CI: 0.73-3.04; p = 0.30 |  |  |  |  |
|  |  |  |  |  | Age-matched Cohort, Multivariate Analysis (Stratified by Holter timing): | |  |  |  |  |
|  |  |  |  |  | Medical management adjusted HR: 2.10; 95% CI: 1.12-3.92; p = 0.02 | Adjusted HR: 1.91; 95% CI: 0.95-3.83; p = 0.07 |  |  |  |  |
| [Ascione, et al. (2023) ^7^](#_ENREF_7) | 63 patients who underwent MV repair | | 14 patients | 11 patients  (5 persistent, 6 new) |  |  |  |  |  |  |
|  | - | PVC/24-hr, median (IQR):  26 (6.5–272.7) |  |  |  |  |  |  |  |  |
|  | - | PVC/24-hr >5%, n (%):  5 (7.9) |  |  |  |  |  |  |  |  |
|  |  |  |  |  | 0.85 ± 3.47 events per patient-month | 0.43 ± 2.03 events per patient-month  (p = 0.01) |  |  |  |  |
|  |  |  |  |  | Anti-tachycardia pacing or Appropriate ICD shock | |  |  |  |  |
|  |  |  |  |  | 1.0 ± 3.87 | 0.32 ± 1.41  (p = 0.014) |  |  |  |  |
| [Pandis, et al. (2024) ^8^](#_ENREF_8) | 30-day freedom from recurrent VA was 100% for m-VA (26/26) and 97.2% for c-VA (35/36) patient subsets (P = 0.395)  The cumulative incidence of recurrent VA at 1 year was 24.1% (n = 13/54) of whom 12 (92.3%; P = .009) were c-VA pre-intervention with preexisting VT.  Overall freedom from recurrent VA at 1 year was 75.9% and greater for m-VA compared with the c-VA subset (95.2% vs 63.6%; P = 0.009) | | | | | | | | | |

Supplemental Table S5: Included case studies investigating surgical repair of MVP, their outcomes/endpoints, and any associations

| Reference | Study Design | Number  (% Female) | Age  (Years) | Study Population | Medical/Surgical Intervention | Diagnostic Criteria | Outcome/Endpoint | Predictor/Association |
| --- | --- | --- | --- | --- | --- | --- | --- | --- |
|  | **Case studies** | | | | | | | |
| [Ross, et al. (1978) ^9^](#_ENREF_9) | Case study | 1 (100%) | 35 | MVP | MVR (#75 Cross-Jones mitral valve prosthesis  Mitral leaflets and papillary muscles were excised | Serial ECGs and Holter | Improvement in clinical symptoms, ECG and Holter findings | NA |
| [Pocock, et al. (1991) ^10^](#_ENREF_10) | Case study | 1 (100%) | 22 | MVP | MVR (34mm Carpentier ring and leaflet excision)  4cm long quadrangular segment of posterior leaflet and triangular segment of anterior leaflet excised | Serial ECGs and Holter | Improvement in clinical symptoms, ECG and Holter findings | NA |
| [Bortnik, et al. (2009) ^11^](#_ENREF_11) | Case study | 1 (100%) | 80 | Cardiac arrest due to VF, secondary to chordae tendinae rupture | Partial quadrangular resection of the medial scallop of the posterior mitral leaflet  The posterior leaflet was sutured (sliding technique)  A complete rigid ring (Saddle Ring n. 32; St Jude Medical, Inc., St Paul, Minnesota, USA) was finally positioned  A single-chamber cardioverter-defibrillator was implanted | Serial ECGs and Holter  TTE | Improvement in clinical symptoms, Holter findings and LV function | NA |
| [Abbadi, et al. (2014) ^12^](#_ENREF_12) | Case study | 1 (100%) | 36 | Bileaflet MVP with moderate mitral regurgitation and normal LV systolic function, cardiac arrest during 4th post-partum month | MV repair with a #36 Edwards Physio Annuloplasty ring  Defibrillator | Defibrillator interrogation during 3-year follow up | Change in VA burden | NA |
| [Hosseini, et al. (2016) ^13^](#_ENREF_13) | Case study | 2 (100%) | 35, 67 | MVP with Refractory VA | Patient 1: P2 triangular resection and sliding plasty of the 2 other posterior scallops. In addition, an AnnuloFlex® annuloplasty ring #34 (Sorin-Carbomedics, Austin, TX, USA) was used to stabilize the mitral ring  Patient 2: Posterior leaflet triangular resection and implantation of a 32-mm Memo 3D annuloplasty ring (Sorin Biomedica Cardio S. R. L., Saluggia, Italy) | Serial ECGs and Holter  TTE | Change in VA burden | NA |
| [Augello, et al. (2017) ^14^](#_ENREF_14) | Case study | 1 (100%) | 46 | Barlow’s disease | MV repair annuloplasty with Cosgrove 32 and neochord implantation | Cardiac MRI  TTE  ECG and Holter | Electrocardiographic and echocardiographic abnormalities post- MVS | NA |
| [Alqarawi, et al. (2018) ^15^](#_ENREF_15) | Case study | 1 (100%) | 39 | Symptomatic severe MR due to bileaflet MVP | MVR and maze procedure  Triangular resection of the posterior leaflet (mid scallop), leaflet plication, and placement of an annuloplasty band (CG future band, Medtronic, Minneapolis, MN) | Serial ECGs and Holter | PVC burden post MVR | NA |
| [Barman, et al. (2021) ^16^](#_ENREF_16) | Case study | 1 (100%) | 42 | Bileaflet MVP, MAD, and severe MR | MV repair  Posterior leaflet quadrangular resection  2 neochordae implanted in the posterior leaflet (P2 and P3)  1 neochordae in the scallops of the anterior leaflet (A1, A2 and A3).  Mitral annuloplasty was applied with a 38-mm Medtronic 3D ring | Holter, ECG and echocardiography prior to and after procedure | Incidence of VA following MVR | NA |

*AF, Atrial Fibrillation; CABG, Coronary Artery Bypass Graft; CAD, Coronary Artery Disease; CRT, Cardiac Resynchronisation Therapy; ECG, Electrocardiogram; EF, Ejection Fraction; HFrEF, Heart failure reduced ejection fraction; ICD, Internal Cardioverter Defibrillator / Implantable Cardiac Device; LA, Left Atrium; LV, Left Ventricle; MAD, Mitral Annular Disjunction; MR, Mitral Regurgitation; MVP, Mitral Valve Prolapse; MVR, Mitral Valve Repair; MVS, Mitral Valve Surgery; PMVR, Percutaneous MVR; PPM, Permanent Pacemaker; PVC, Premature Ventricular Complex; SD, Sudden death; TTE, Transthoracic Echocardiogram; VA, Ventricular Arrhythmia; VE, Ventricular Ectopic; VF, Ventricular Fibrillation; VT, Ventricular Tachycardia;*

Table S6: Included case studies, study populations, length of follow-up, and whether there was a reduction in VA or SCD post- MVS or pMVR

| Reference | Medical/Surgical Intervention | Study population | Follow-up | Change in VA burden or SCD following intervention | | | | |
| --- | --- | --- | --- | --- | --- | --- | --- | --- |
|  | **MVS (repair / replacement)** | | | | | | | |
|  | **Case Studies** | | | | | | | |
| [Ross, et al. (1978) ^9^](#_ENREF_9) | MV replacement with #75 Cross-Jones MV prosthesis | 35-year-old female, BiMVP with myxomatous degeneration, aborted SCD | First seen age 24 years old in 1966  MV replacement 1974  3.5 years follow-up until 1978 | ↓PVC |  | ↓VT |  | - |
| [Pocock, et al. (1991) ^10^](#_ENREF_10) | MV repair, and annuloplasty with a 34 mm Carpentier ring | 22-year-old female, MVP (billowing of the posterior leaflet and mild MR) | 22 years pre-surgery  21 months post-surgery | ↓PVC |  |  |  | - |
| [Bortnik, et al. (2009) ^11^](#_ENREF_11) | Partial quadrangular resection of medial scallop, and the posterior leaflet was sutured (sliding technique)  Annuloplasty with complete rigid ring (Saddle Ring #32 St Jude Medical, Inc.,  St Paul, Minnesota, USA) | 80-year-old female, cardiac arrest due to VF secondary to chordae tendinae rupture | 16 months post- surgery and ICD implantation |  |  |  | ↓VF  (Absence of any VA) | - |
| [Abbadi, et al. (2014) ^12^](#_ENREF_12) | MV repair, and annuloplasty with #36 Edwards Physio ring | 36-year-old female, BiMVP with moderate MR and normal LV systolic function, cardiac arrest during 4th post-partum month | 3 years post-surgery | No episodes of NSVT post-operatively, no ICD shocks and no palpitations | |  |  | - |
| [Hosseini, et al. (2016) ^13^](#_ENREF_13) | Patient 1: MV repair with P2 triangular resection and sliding plasty of the 2 other posterior scallops, and annuloplasty ring.  Patient 2: MV repair with posterior triangular resection, and annuloplasty ring. | Patient 1: 35-year-old female, severe MR with BiMVP  Patient 2: 67-year-old female, flail posterior MV leaflet with severe MR, aborted SCD | Patient 1: 18 months post-surgery  Patient 2: 3.5 years post-surgery | ↓PVC | ↓NSVT | - | - | - |
| [Augello, et al. (2017) ^14^](#_ENREF_14) | MV repair (annuloplasty with Cosgrove #32, neochord implantation) | 46-year-old female with Barlow’s disease (MVP diagnosed aged 20) and significant MAD | 3 months post-surgery | ↓PVC | - | - | - | - |
| [Alqarawi, et al. (2018) ^15^](#_ENREF_15) | MV repair and maze procedure  Triangular resection of the posterior leaflet (mid scallop), leaflet plication, and placement of an annuloplasty band | 39-year-old female, symptomatic severe MR due to myxomatous BiMVP | 6 months post-surgery | ↓PVC | - | - | - | - |
| [Barman, et al. (2021) ^16^](#_ENREF_16) | Posterior leaflet quadrangular resection, with 2 neochordae implanted in the posterior leaflet (P2 and P3), and 1 neochordae in the scallops of the anterior leaflet (A1, A2 and A3).  Mitral annuloplasty was applied with a 38-mm Medtronic 3D ring | 42-year-old female, BiMVP, MAD, severe MR, LGE inferolateral LV wall | 6 months post-surgery | - | ↓NSVT | - | - | - |

*BiMVP, Bileaflet MVP; ECG, Electrocardiogram; HFrEF, Heart Failure reduced Ejection Fraction; ICD, Internal Cardioverter Defibrillator / Implantable Cardiac Device; LA, Left Atrium; LV, Left Ventricle; LVEF, LV Ejection Fraction; MAD, Mitral Annular Disjunction; MR, Mitral Regurgitation; MVP, Mitral Valve Prolapse; MVR, Mitral Valve Repair; MVS, Mitral Valve Surgery; NYHA, New York Heart Association; pMVR, percutaneous MV repair; PVC, Premature Ventricular Complex; SCD, Sudden Cardiac death; VA, Ventricular Arrhythmia; VF, Ventricular Fibrillation; VT, Ventricular Tachycardia*

*Table S7: Effect of mitral intervention on arrhythmic outcomes in MVP – A Risk of bias assessment using the ROBINS-I (The Risk Of Bias In Non-randomized Studies – of Interventions) tool for each included cohort study*


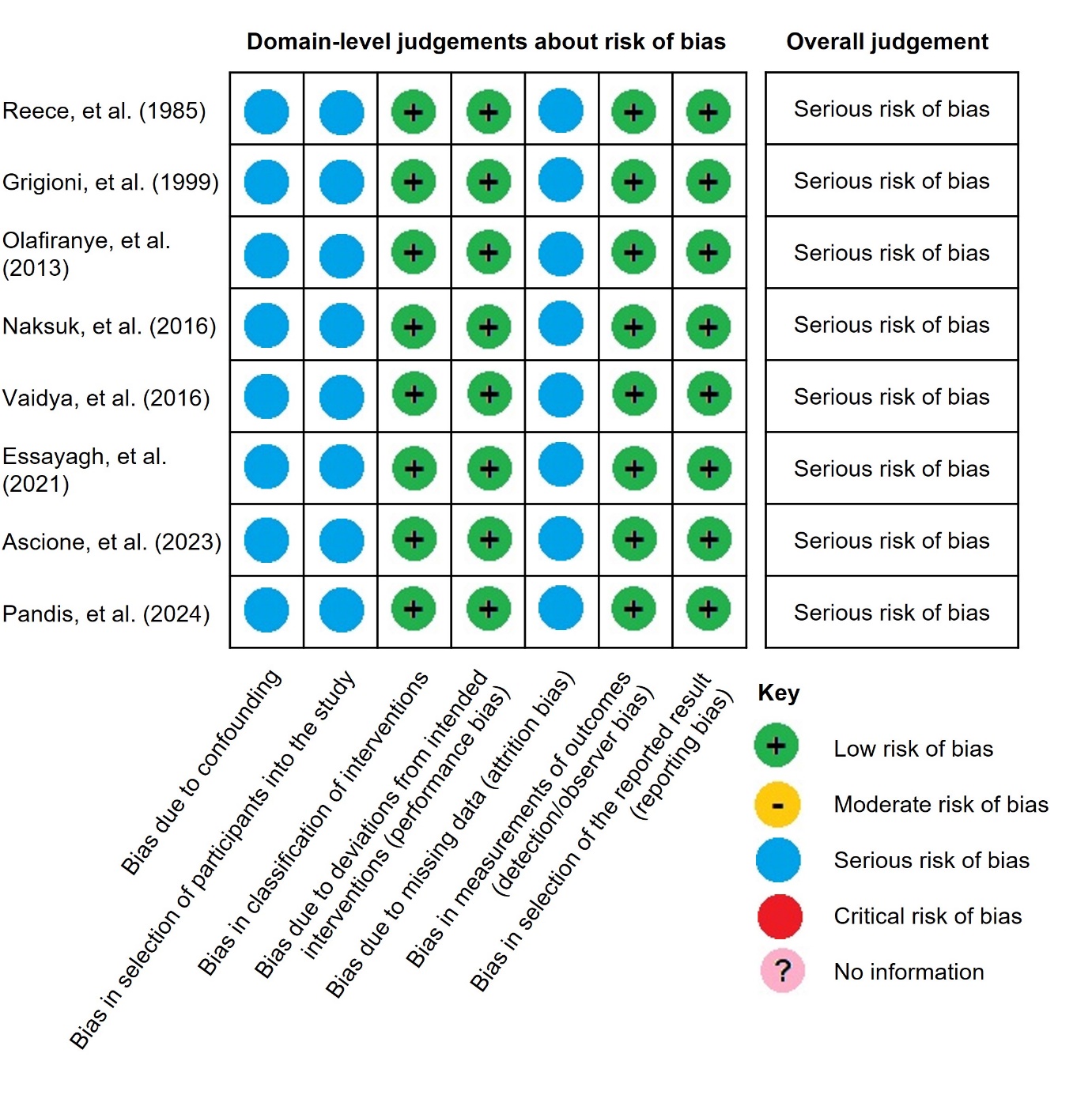


# References

1. Reece IJ, Cooley DA, Painvin GA, et al. Surgical treatment of mitral systolic click syndrome: results in 37 patients. *The Annals of thoracic surgery*. 1985;39:155-158. doi: 10.1016/s0003-4975(10)62556-8

2. Grigioni F, Enriquez-Sarano M, Ling LH, et al. Sudden death in mitral regurgitation due to flail leaflet. *Journal of the American College of Cardiology*. 1999;34:2078-2085. doi: 10.1016/s0735-1097(99)00474-x

3. Olafiranye O, Hochreiter CA, Borer JS, et al. Nonischemic mitral regurgitation: prognostic value of nonsustained ventricular tachycardia after mitral valve surgery. *Cardiology*. 2013;124:108-115. doi: 10.1159/000347085

4. Naksuk N, Syed FF, Krittanawong C, et al. The effect of mitral valve surgery on ventricular arrhythmia in patients with bileaflet mitral valve prolapse. *Indian pacing and electrophysiology journal*. 2016;16:187-191. doi: 10.1016/j.ipej.2016.10.009

5. Vaidya VR, DeSimone CV, Damle N, et al. Reduction in malignant ventricular arrhythmia and appropriate shocks following surgical correction of bileaflet mitral valve prolapse. *Journal of interventional cardiac electrophysiology : an international journal of arrhythmias and pacing*. 2016;46:137-143. doi: 10.1007/s10840-015-0090-5

6. Essayagh B, Sabbag A, Antoine C, et al. The Mitral Annular Disjunction of Mitral Valve Prolapse. *JACC Cardiovascular imaging*. 2021;14:2073–2087. doi: 10.1016/j.jcmg.2021.04.029

7. Ascione G, Azzola Guicciardi N, Lorusso R, et al. The impact of mitral valve surgery on ventricular arrhythmias in patients with Barlow's disease: preliminary results of a prospective study. *Interdiscip Cardiovasc Thorac Surg*. 2023;36. doi: 10.1093/icvts/ivad073

8. Pandis D, David N, Ei-Eshmawi A, et al. Noncomplex ventricular arrhythmia associated with greater freedom from recurrent ectopy at 1 year after mitral repair surgery. *JTCVS Open*. 2024;19:94-113. doi: 10.1016/j.xjon.2024.04.005

9. Ross A, DeWeese JA, Yu PN. Refractory ventricular arrhythmias in a patient with mitral valve prolapse. Successful control with mitral valve replacement. *Journal of electrocardiology*. 1978;11:289-295. doi: 10.1016/s0022-0736(78)80131-9

10. Pocock WA, Barlow JB, Marcus RH, et al. Mitral valvuloplasty for life-threatening ventricular arrhythmias in mitral valve prolapse. *American heart journal*. 1991;121:199-202. doi: 10.1016/0002-8703(91)90976-o

11. Bortnik M, Leverone M, Teodori G, et al. Ventricular fibrillation in acute mitral valve insufficiency caused by chordae tendineae rupture: report of a surgically corrected case. *Journal of cardiovascular medicine (Hagerstown, Md)*. 2009;10:261-263. doi: 10.2459/jcm.0b013e3283207b6f

12. Abbadi DR, Purbey R, Poornima IG. Mitral valve repair is an effective treatment for ventricular arrhythmias in mitral valve prolapse syndrome. *International journal of cardiology*. 2014;177:e16-18. doi: 10.1016/j.ijcard.2014.07.174

13. Hosseini S, Rezaei Y, Samiei N, et al. Effects of mitral valve repair on ventricular arrhythmia in patients with mitral valve prolapse syndrome: A report of two cases. *International journal of cardiology*. 2016;222:603-605. doi: 10.1016/j.ijcard.2016.08.053

14. Augello M, Lanzarini L. Disappearance of Electrocardiographic Abnormalities Associated with the Arrhythmic Pattern of a Barlow Disease After Surgical Mitral Valve Repair. *Clinics and practice*. 2017;7:946. doi: 10.4081/cp.2017.946

15. Alqarawi W, Birnie DH, Burwash IG. Mitral valve repair results in suppression of ventricular arrhythmias and normalization of repolarization abnormalities in mitral valve prolapse. *HeartRhythm case reports*. 2018;4:191-194. doi: 10.1016/j.hrcr.2018.02.012

16. Barman HA, Yildiz A, Güden M, et al. The disappearance of nonsustained ventricular tachycardia after surgical repair in a patient with mitral annular disjunction and mitral valve prolapse. *Turk Kardiyoloji Dernegi arsivi : Turk Kardiyoloji Derneginin yayin organidir*. 2021;49:419-423. doi: 10.5543/tkda.2021.04501
